# Glycerol Carbonate: A Novel Biosolvent with Strong Ionizing and Dissociating Powers

**DOI:** 10.1100/2012/697161

**Published:** 2012-05-02

**Authors:** Guangnan Ou, Biyan He, Xuejing Li, Jianhui Lei

**Affiliations:** School of Bioengineering, Jimei University, Xiamen 361021, China

## Abstract

The activity of biocatalysts in nonaqueous solvents is related to the interaction of organic solvents with cells or enzymes. The behavior of proteins is strongly dependent on the protonation state of their ionizable groups, which ionization constants are greatly affected by the solvent. Due to the weak ionizing and dissociating powers of common organic solvents, the charge of the protein will change significantly when the protein is transferred from water to common organic solvents, resulting in protein denaturation. In this work, glycerol carbonate (GC) was synthesized, which ionizing and dissociating abilities were very close to those of water. Transesterification activities of *Candida antarctica* lipase B (CALB) in GC were comparable to those in water and remained constant during 4-week storage. *Bacillus subtilis* and *Saccharomyecs cerevisiae* were cultured in liquid media containing GC with test tubes. In the medium containing low GC concentration, *Bacillus subtilis* and *Saccharomyecs cerevisiae* grew well as in a medium containing no organic solvent, but, in the medium containing high GC concentration, the growth of *Bacillus subtilis* and *Saccharomyecs cerevisiae* was suppressed. The results suggested that GC is a potential biosolvent, which has great significance to biocatalysis in nonaqueous solvents.

## 1. Introduction

The activity of biocatalysts in nonaqueous solvents is related to the interaction of the solvents with cells or enzymes [1–4]. At the molecular level, solvents can exert toxicity towards cells via enzyme inhibition, protein denaturation, and membrane modifications. Although the current view of organic solvent cytotoxicity focuses mainly on the interaction of these molecules with membrane lipids [5–7], we should recognize the importance of proteins as the targets of organic solvent in intact cells.

The behavior of proteins is strongly dependent on the protonation state of their ionizable groups [8–10]. The ionization constant of weak electrolyte is greatly affected by the solvent. In order to take full advantage of the effects of different solvents on enzyme function in designing reaction systems, an understanding of the molecular interactions between solvent and protein is essential. For the ionizable groups (AB) of an enzyme, the ionization process can be broken conceptually into two steps [11, 12]:

(1) $$\underset{\left(\text{electrolyte}\right)}{{\left(\text{AB}\right)}_{\text{solv}}}\overset{\text{Ionization}}{⇌}\underset{\left(\text{ion}\;\text{pair}\right)}{{\left({\text{A}}^{+\;},\;{\text{B}}^{-}\right)}_{\text{solv}}}\overset{\text{Dissociation}}{⇌}\underset{\left(\text{free}\;\text{solvated}\;\text{ions}\right)}{{\left({\text{A}}^{+}\right)}_{\text{solv}}+\;{\left({\text{B}}^{-}\right)}_{\text{solv}}}.$$

In the first step, solvent molecules act as a Lewis base (electron pair donor (EPD)) to the A atom and as a Lewis acid (electron pair acceptor (EPA)) to the B atom and ionize the A and B atoms, resulting in the breaking of the A–B covalent bond and the formation of the ion pair (A^+^,B^−^)_solv_. The ionization becomes easy if solvent molecules interact strongly with the A and B atoms. The ionization step is a function of the donor number (DN) and acceptor number (AN) of the solvent. Higher values mean that solvent has higher ability to ionize neutral molecules and to stabilize the formed ions. In the second step, the ion pair (A^+^,B^−^)_solv_ dissociates into free ions. The dissociation process is easy if the solvent has a high dielectric constant (*ε*_*r*_) and the distance of the closest approach between A^+^ and B^−^ is large enough. Thus, two important solvent properties, that is, dielectric constant and donor-acceptor properties, significantly influence both ionization and dissociation processes.

**Figure 1:**
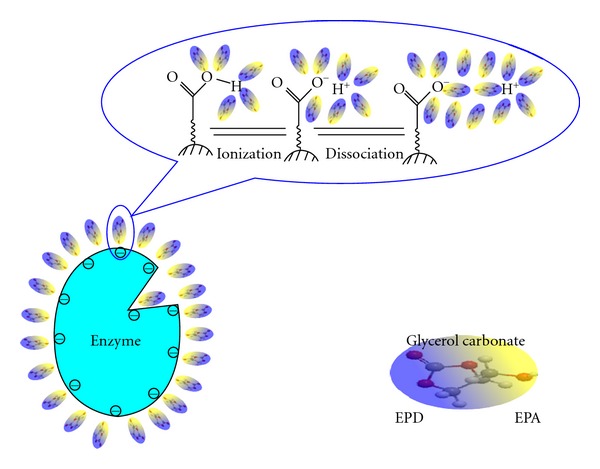
Ionization and dissociation processes of an enzyme in GC.

On the basis of the above analysis, we recently constructed a new class of biosolvents with cyclic carbonate moiety [10]. We synthesized glycerol carbonate (GC) for the development of soluble enzymatic systems and investigated the effects of various reaction parameters on activity and stability of soluble enzymes. The results showed that GC is a good ionizing and dissociating solvent and transesterification activities of *Candida antarctica* lipase B (CALB) and *Candida rugosa* lipase (CRL) in GC are similar to those in water. The water-like activities of lipases in GC can be explained by the viewpoint of ionization. Like water, GC has higher ability to ionize neutral ionizable groups of an enzyme and to stabilize the formed ions. In addition, with its high polarity GC is capable of reducing the strong electrostatic attraction between oppositely charged ions to such an extent that ion pairs can dissociate into free solvated ions (Figure 1). Thus, the ionization state and active conformation of the enzyme are maintained when the enzyme is transferred from water to GC.

To obtain more information on the reasons for the improvement of enzyme efficiency in GC, we investigated long-term storage stability of CALB in GC and the structure-stability relationships of CALB by mass spectrometry. The knowledge gained in nonaqueous enzymatic catalysis was then applied to the cultivation of microorganisms in nonaqueous media.

## 2. Materials and Methods

### 2.1. Materials and Strains

*Candida antarctica* lipase B (CALB, ~9 U mg^−1^) was purchased from Sigma and used as supplied. Ethyl butyrate and 1-butanol were analytical reagents and were dried by oven-dried (400°C) 3A molecular sieves before use. All other chemicals and reagents were of analytical grade.

*Bacillus subtilis* and *Saccharomyecs cerevisiae* were from the School of Bioengineering, Jimei University.

### 2.2. Synthesis of Glycerol Carbonate

Glycerol carbonate (GC) was synthesized according to published procedures [10]. Colorless liquid was obtained. The solvent was stored over oven-dried (400°C) 3A molecular sieves at least 24 h prior to use. ^1^H NMR (400 MHz, D_2_O) *δ*: 4.927–4.980 (m, 1H, –CH–), 4.63 (t, 1H, –OCH_2_–, J1 = 8.67 Hz, J2 = 8.67 Hz), 4.39 (dd, 1H, –OCH_2_–, J1 = 6.20 Hz, J2 = 8.67 Hz), 3.79 (ddd, 2H, –CH_2_OH, J1 = 3.29 Hz, J2 = 13.19 Hz, J3 = 17.18 Hz); ^13^C NMR (400 MHz, D_2_O) *δ*: 157.767, 78.101, 66.885, 60.973.

### 2.3. Characterizations

The ^1^H NMR and ^13^C NMR spectra were obtained on a Brüker AV-400 Fourier transform NMR spectrometer. ^1^H NMR spectra were referenced to tetramethylsilane.

The mass spectra were acquired using a Microflex (Bruker Daltonics, Bremen, Germany) time-of-flight (TOF) mass spectrometer. The spectra were acquired in positive ion mode in the range m/z 1000–100000. 3,5-dimethoxy-4-hydroxycinnamic acid (20 mg, Aldrich) was dissolved in 1 mL methanol/acetonitrile 50 : 50 (v/v) and used as matrix. Aliquots (5 *μ*L) of each enzyme sample (2 mM) were mixed with an equivalent aliquot of the matrix solution; samples of 1 *μ*L of the resulting solutions were laid on the stainless steel target and allowed to dry under a nitrogen flow at room temperature. MALDI-TOF MS experiments were performed immediately after solvent evaporation.

### 2.4. General Procedures of Enzymatic Transesterification

Lipase powder (1.2 mg) was dissolved in 500 *μ*L of solvent in a 5 mL flask and incubated at room temperature (20°C). At the time points indicated, 110 *μ*L (0.83 mmol) ethyl butyrate, 110 *μ*L (1.21 mmol) 1-butanol, and 50 *μ*L nonane (internal standard) were added. The reaction mixture was stirred at 40°C in oil bath. After 30 minutes of reaction, a 100 *μ*L aliquot of the reaction mixture was added to one portion of water and extracted with two portions of cyclohexane three times. The combined organic phase was analyzed with a gas chromatograph equipped with an FID and a capillary column (SE-30, 30 m × 0.32 mm × 0.25 *μ*m).

### 2.5. Preparation of Culture Media

Luria-Bertani (LB) media consisted of 0.5% (w/v) yeast extract, 1% (w/v) tryptone, and 1% (w/v) NaCl in distilled water. For the preparation of YPD media, 1% (w/v) yeast extract and 2% (w/v) peptone were dissolved in distilled water and autoclaved. Afterwards sterile D-glucose (2% w/v) was added to give 0.2% (w/v).

### 2.6. Growth of *Bacillus subtilis* and *Saccharomyecs cerevisiae*

Inocula were prepared by transferring loopfuls of pure culture in media (LB or YPD media) followed by incubation: yeast* Saccharomyecs cerevisiae* was incubated in YPD media at 30°C and 200 rpm and *Bacillus subtilis* strain in LB media at 37°C and 200 rpm. After the absorbance at 600 nm (A_600_) of cultures reached 0.4-0.5, 0.1 mL of the preculture was inoculated into 5 mL fresh media with varying amounts of GC in new sterile tubes, followed by incubation. Growth was measured as a function of A_600_.

## 3. Results and Discussion

### 3.1. Long-Term Storage Stability of CALB in GC

**Figure 2:**
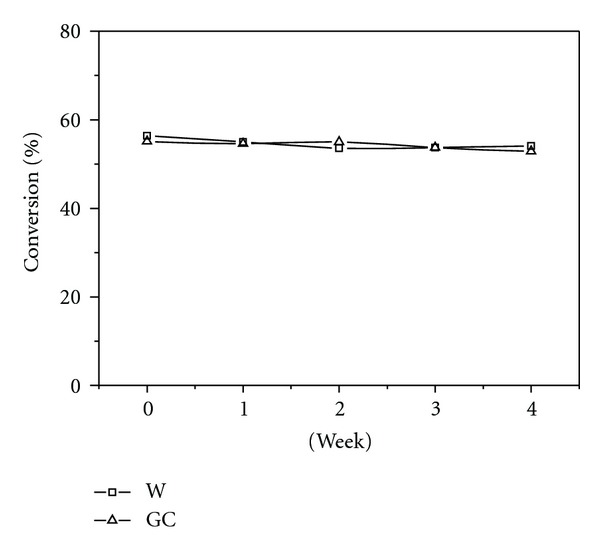
Storage stability of CALB in water and GC. Reaction conditions: Lipase powder (1.2 mg); 500 *μ*L of solvent; 110 *μ*L of ethyl butyrate (0.83 mmol); 110 *μ*L of 1-butanol (1.21 mmol); stirring speed = 300 rpm; temperature = 40°C. Conversions refer to ethyl butyrate.

In a previous study of ours [10], in which we compared the effects of temperature and water content on the activity of CALB in GC and in water, we found that CALB showed an activity in GC close to that displayed in water. To obtain more information on the reasons for the improvement of enzyme efficiency in GC, we investigated long-term storage stability of CALB in GC. The stability of an enzyme is of great importance for its commercial use. To elucidate the effect of prolonged exposure of CALB in GC, enzyme solutions (dissolved enzyme) were incubated under air in closed vessels at 20°C up to 4 weeks. Residual activity was measured in standard test using the transesterification of ethyl butyrate with 1-butanol as an enzyme activity test. The results were also compared with those obtained in water medium. As can be seen in Figure 2, CALB in GC is as stable as in water during long-term storage: its residual activity after incubation in GC upon air within 4 weeks remained at the 100% of its activity during this period. 

The water-like activities of lipases in GC (log⁡  *P* = −0.25) [10] cannot be explained by the rule proposed by Laane et al. [13], which states that hydrophilic solvents (log⁡  *P* < 2) are not suitable for enzymatic catalysis. From the viewpoint of ionization, the ionization constant of ionizable groups is greatly affected by the solvent. It was reported that the pK_a_ values of weak electrolytes are significantly higher in common organic solvents than in water [11, 12]. Therefore, the charge of the enzyme will change significantly when the enzyme is transferred from water to common organic solvents. It was observed that changing the surface charge of subtilisin by site-directed mutagenesis produces enzymes with significantly shifted pH-activity profile, higher catalytic activity [14], and higher stability [15]. Based on the above analyses, common organic solvents affect not only the ionization state of active site groups of the enzyme but also electrostatic interactions within the protein, and, ultimately, denaturation occurs. In the case of GC, its ionizing and dissociating abilities are similar to water [10], and, thus, the ionization state of the enzyme is maintained when the enzyme is transferred from water to GC. Therefore, transesterification activities of *Candida antarctica* lipase B (CALB) in GC were comparable to those in water and remained constant during storage, indicating that the newly designed compound is a potential biosolvent.

### 3.2. Structure-Stability Relationships of CALB by Mass Spectrometry

**Figure 3:**
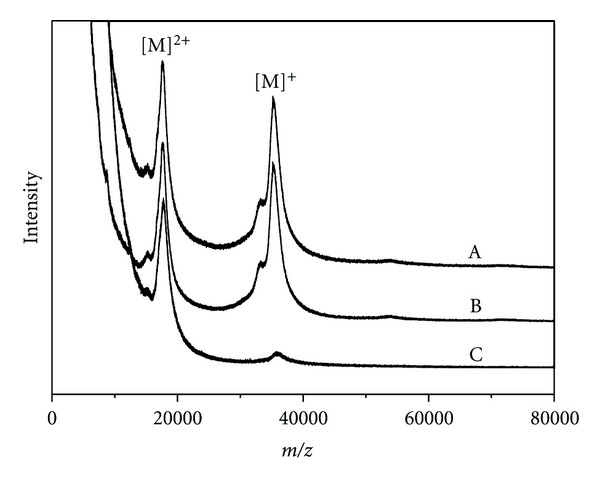
MALDI-TOF MS-positive ion spectra of CALB in GC. A. fresh sample; B. sample after 4 weeks storage; C. sample heated at 96°C for 10 min.

Mass spectrometry (MS) has emerged as an extremely useful technique for the analysis of a wide variety of biological samples. Some of the advantages of MALDI-MS include a relatively simple sample preparation, high sensitivity (low femtomole range), and rapid analysis times. Typically, only singly, doubly, or triply charged molecular ions are observed, which facilitates spectral interpretation. As shown in Figure 3, the singly and doubly charged molecular ions are the base peaks. The spectra profiles before and after incubation of CALB in GC at room temperature were similar in molecular weight and in intensity. In comparison with the charge state distributions of the protein formed from denaturing solution condition (incubated at 96°C for 10 min, curve C in Figure 3), which are shifted to higher charge states (lower m/z) than those formed from solutions incubated at room temperature (curve A and curve B in Figure 3), it is obvious that CALB in GC has significant tertiary structure at room temperature. Our results are consistent with the conclusions reported by literatures [16–18].

Protein function almost always involves rapid, reversible changes in protein conformation [19]. To allow these structural changes to occur rapidly, proteins can be neither too rigid nor too flexible. The conformational rigidity is the result of noncovalent interactions which are essentially of the electrostatic origin [20]. According to Coulomb's law, the strength of the interactions is inversely proportional to the dielectric constant. Therefore, enzymes are much more rigid in solvents of low dielectric constant than in those of high dielectric constant. Because the dielectric constant of GC (*ε* = 82.66) is close to that of water (*ε* = 78.30) [10], CALB has the same conformational flexibility in GC as in water. Consequently, transesterification activities of CALB in GC were comparable to those in water and remained constant during storage. These results show the excellent ability of GC to maintain an active conformation of the enzyme and to provide suitable microenvironments. The combination of the storage stability and MALDI-TOF MS results indicated that CALB dissolved in GC is very active and stable. The results, once again, showed that GC is a potential biosolvent.

### 3.3. Cultivation of Microbes in Glycerol Carbonate

**Figure 4:**
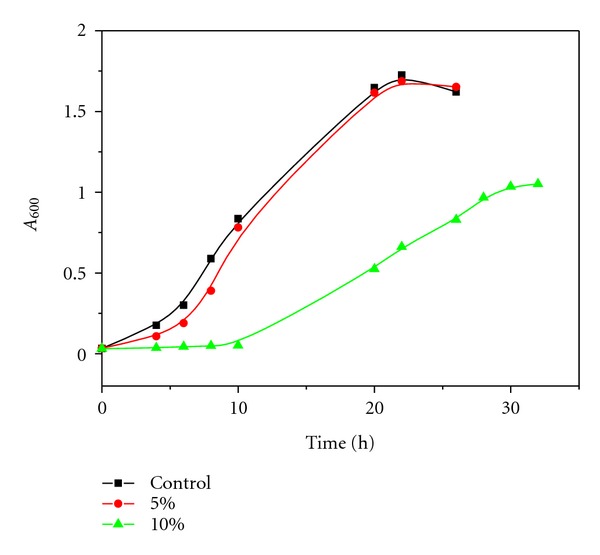
Growth curves of *Bacillus subtilis* in a medium with varying percentage of GC (% v/v).

**Figure 5:**
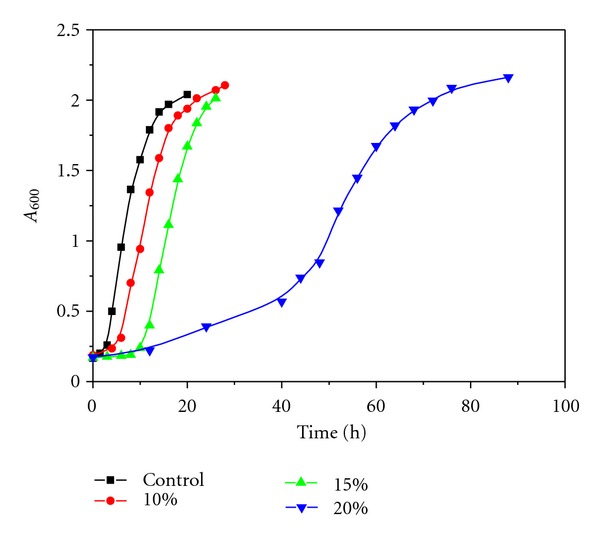
Growth curves of *Saccharomyecs cerevisiae* in a medium with varying percentage of GC (% v/v).

Inspired by the success in enzymatic catalysis in GC, we extended the study to the cultivation of microbes in glycerol carbonate. Since Gram-negative bacteria have an additional outer membrane made up of phospholipids and lipopolysaccharides compared to the single cytoplasmic membrane of Gram-positive bacteria, it was assumed that Gram-negative bacteria are better equipped to cope with solvent-induced shock. Effects of nonpolar organic solvents particularly in Gram-negative bacteria were studied in great detail [21–25]. In contrast, studies developed using polar organic solvents in Gram-positive microorganisms are very scarce. We, therefore, chose *Bacillus subtilis *as a model bacterium. As for yeast, *Saccharomyecs cerevisiae* has developed as a model organism because it shares the complex internal cell structure of plants and animals without the high percentage of noncoding DNA that can confound research in higher eukaryotes. In this context, *Bacillus subtilis* and *Saccharomyecs cerevisiae* were cultured in liquid media containing GC with test tubes. As can be seen in Table 1, in the medium containing low GC concentration, *Bacillus subtilis* and *Saccharomyecs cerevisiae* grew well as in a medium containing no organic solvent, but in the medium containing high GC concentration, the growth of *Bacillus subtilis* and *Saccharomyecs cerevisiae* was suppressed. We then focus on the characteristics of microbial growth in the medium containing low GC concentration. The results were shown in Figures 4 and 5. It can be seen that the length of the lag period varied from one population to another, depending on the condition of the microbes and GC concentration. Growth lags primarily because the newly inoculated cells require a period of adjustment, enlargement, and synthesis of DNA, enzymes, and ribosomes.

Although GC is a suitable solvent for nonaqueous enzymatic catalysis, it can suppress the growth of microbes. At the molecular level, solvents can exert toxicity towards cells via enzyme inhibition, protein denaturation, and membrane modifications such as membrane expansion, structure disorders, and permeability changes due to the accumulation of the solvent in the membrane [24, 26]. From experimental results of storage stability and MALDI-TOF MS results, the increase in ionizing and dissociating abilities is expected to result in a decreased molecular toxicity effect. With its strong ionizing and dissociating abilities, GC is able to prevent protein denaturation. In fact, in the medium containing low GC concentration, *Bacillus subtilis* and *Saccharomyecs cerevisiae* grew well as in a medium containing no organic solvent. In the medium containing high GC concentration, however, the growth of *Bacillus subtilis* and *Saccharomyecs cerevisiae* was suppressed. It is probable that GC may accumulate in the membrane and disrupt the lipid bilayer, thus compromising cell viability. It has been proved that the greater the polarity, the lower the log *P* value and the greater the degree of accumulation of the solvent in the membrane [27, 28].

It is highly unlikely that a single characteristic will be able to predict the biocompatibility of solvents. It is thus probable that the further addition of a number of carefully chosen characteristics will be able to improve the system further. As one future goal, we will focus in particular on the design of a new class of organic solvents with both strong ionizing-dissociating abilities and proper polarity.

## 4. Conclusions

In summary, glycerol carbonate (GC) was synthesized, which ionizing and dissociating abilities were very close to those of water. Transesterification activities of *Candida antarctica* lipase B (CALB) in GC were comparable to those in water and remained constant during 4 weeks of storage. The mass spectra profiles before and after 4 weeks of incubation of CALB in GC at room temperature were similar in molecular weight and in intensity, indicating that the tertiary structure of CALB was maintained after incubation at room temperature. *Bacillus subtilis* and *Saccharomyecs cerevisiae* were cultured in liquid media containing GC with test tubes. In the medium containing low GC concentration, *Bacillus subtilis* and *Saccharomyecs cerevisiae* grew well as in a medium containing no organic solvent, but in the medium containing high GC concentration, the growth of *Bacillus subtilis* and *Saccharomyecs cerevisiae* was suppressed. It is highly unlikely that a single characteristic will be able to predict the biocompatibility of solvents. As one future goal, we will focus in particular on the design of a new class of organic solvents with both strong ionizing-dissociating abilities and proper polarity.

## Figures and Tables

**Table 1:** Effect of GC concentration on growth of microbes.

C_GC_/% v/v	A_600_	
	*Bacillus subtilis*	*Saccharomyecs cerevisiae*
0	1.457	2.081
5	1.410	—
10	0.029	1.853
15	0.002	1.764
20	0	0.068
40	0	0

Cultivation conditions: incubation time: 24 h; incubation temperature: 37°C for *Bacillus subtilis* and 30°C for *Saccharomyecs cerevisiae*.

## Abbreviations

CALB: *Candida antarctica* lipase B
CRL: *Candida rugosa* lipase
GC: Glycerol carbonate
EPD: Electron pair donor
EPA: Electron pair acceptor
DN: Donor number
AN: Acceptor number.
